# Comparative Study of Porous Iron Foams for Biodegradable Implants: Structural Analysis and In Vitro Assessment

**DOI:** 10.3390/jfb14060293

**Published:** 2023-05-24

**Authors:** Gabriela Gąsior, Marlena Grodzicka, Tomasz Jędrzejewski, Marek Wiśniewski, Aleksandra Radtke

**Affiliations:** 1 Faculty of Chemistry, Nicolaus Copernicus University in Toruń, Gagarina Street 7, 87-100 Toruń, Poland; m.grodzicka@doktorant.umk.pl (M.G.); marek.wisniewski@umk.pl (M.W.); 2 Faculty of Biological and Veterinary Sciences, Nicolaus Copernicus University in Toruń, Lwowska Street 1, 87-100 Toruń, Poland; tomaszj@umk.pl

**Keywords:** iron, iron-based materials, porosity, biodegradable metals, corrosion, cytotoxic activity

## Abstract

Biodegradable metal systems are the future of modern implantology. This publication describes the preparation of porous iron-based materials using a simple, affordable replica method on a polymeric template. We obtained two iron-based materials with different pore sizes for potential application in cardiac surgery implants. The materials were compared in terms of their corrosion rate (using immersion and electrochemical methods) and their cytotoxic activity (indirect test on three cell lines: mouse L929 fibroblasts, human aortic smooth muscle cells (HAMSC), and human umbilical vein endothelial cells (HUVEC)). Our research proved that the material being too porous might have a toxic effect on cell lines due to rapid corrosion.

## 1. Introduction

Medical implants have been the subject of intensive research in recent years, resulting in the development of numerous materials used in various medical fields. Biodegradable metals, particularly for bone and cardiovascular implants, have gained significant popularity. These metals are recognized for their ability to slowly degrade through corrosion within the body, offering temporary mechanical support and eventual replacement by regenerated tissue [1]. This property presents several advantages, including reduced complications and the necessity for revision surgery, the avoidance of long-term anticoagulation issues, and the elimination of CT/MRI imaging artifacts. As a result, they hold great promise as an alternative to traditional metal implants [2,3,4].

Zinc, magnesium, and iron are the most promising biodegradable metals for medical applications. Zinc has been extensively studied in medical implants due to its biocompatibility and ability to promote tissue growth. It has been shown to have antibacterial properties and can help reduce inflammation, making it particularly useful for orthopedic and dental implants. Additionally, zinc has a low toxicity level and is readily available, making it an attractive material for medical devices. However, one of the main disadvantages of zinc is its relatively short degradation time and poor mechanical properties, which can limit its use in long-term implantation [5,6]. Magnesium is another biodegradable metal that has been extensively studied for implant applications. Magnesium has a high strength-to-weight ratio, which makes it a suitable candidate for orthopedic applications. It degrades via corrosion, producing magnesium ions, which can stimulate bone growth. However, the rapid degradation of magnesium can lead to excessive hydrogen production, which causes inflammation in the surrounding tissues. Efforts are underway to develop coatings and alloys that can modulate the corrosion rate and improve mechanical properties [7,8]. Iron is a vital element in the body, and its bioabsorbable nature makes it a perfect candidate for medical implants. It degrades via corrosion, and its corrosion products, such as ferrous and hydroxide ions, are biologically compatible. The corrosion rate of iron is relatively slow, which could be a potential limitation. However, it is worth considering trying to accelerate the corrosion of iron via surface modifications or doping with other elements, since iron is biocompatible and has excellent mechanical properties, including Young’s modulus, hardness, and tensile strength [9,10].

Biodegradable metals have shown great promise for medical implants, especially bone implants and cardiac heart valves. The choice of biodegradable metal should consider its advantages and disadvantages, including its corrosion rate, mechanical properties, and biocompatibility. The material’s structure, morphology, and porosity should also be optimized to improve the implant’s performance [11]. Porous materials enhance cell growth and facilitate the ingrowth of new tissue. The porous structure also helps change the degradation rate and prolong the implant’s mechanical stability. The morphology of the implant can also impact its mechanical properties, and research is ongoing to develop implant geometries that can maximize the biological response while maintaining the mechanical properties [10,12].

In this article, we describe a porous material that is formed from pure iron using a polymer as a template via the replica method, which is elaborated on in Section 2.2. The utilization of polymers in this method is advantageous due to their ease of production, ability to form complex shapes, cost-effectiveness, and widespread availability.

By utilizing two distinct templates, we were able to create materials with varying morphology and structure, enabling a comparative analysis of the corrosion rate, cytotoxicity, and surface energy.

## 2. Materials and Methods

### 2.1. Materials

The materials presented in this work are iron foams, differing in porosity, depending on the polymer template used. They were prepared from iron powder with a particle size of <10 microns (purity 99.9%, Alfa Aesar, Kandel, Germany), and two commercially available polymer foams. These werepolyurethane (PU PPI15 Rekuperator, Wejherowo, Poland), with large pores, and a melamine sponge (10 × 6.2 × 2.8 cm, Polor Sp z o.o., Szczecin, Poland), with visibly smaller pores. The system prepared on the template and made on the polymer foam is later referred to as Fe01, while the system on the melamine foam is known as Fe02, in order to make the text legible and easier to read.

### 2.2. Preparation of Iron Foams

**Figure 1:**
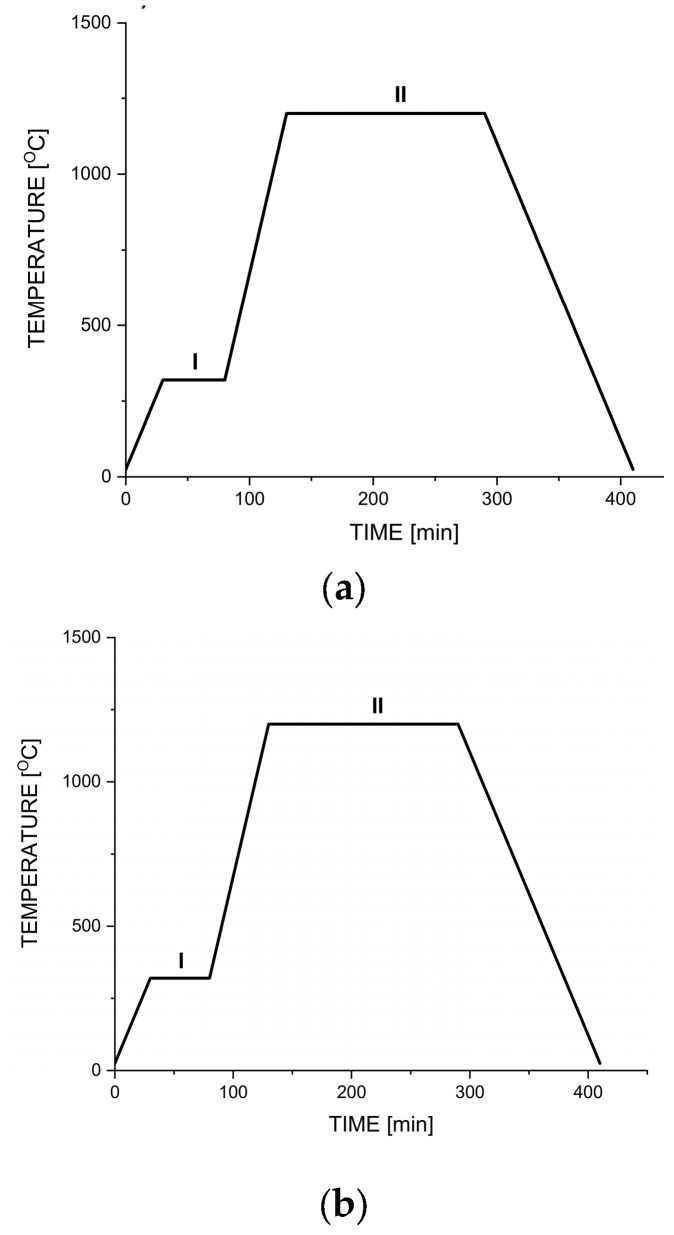
Scheme of the used heat treatment—temperature program; Two most essential processes: I—removal of the polymer template with the flow of gases; II—sintering of iron powder while preserving the template’s structure. (**a**) Process for Fe01; (**b**) Process for Fe02.

Depending on the needs of the method, the sponges were adequately shaped and then soaked in a solution of 5% polyvinyl alcohol (Mw 89,000–98,000, 99+% hydrolyzed, Sigma Aldrich, Darmstadt, Germany), mixed previously with iron powder (mass ratio 1:2). Then, the foams were dried in a laboratory dryer for 35 min at 95 °C, placed in a tube furnace (Czylok, Jastrzębie-Zdrój, Poland), and sintered in the reductive atmosphere, according to the diagrams shown in Figure 1. Both technological processes were adapted to the properties of the polymer used as a template.

After the synthesis, all samples were always ultrasonically cleaned in acetone (ACS reagent, >99.5% Sigma Aldrich, Darmstadt, Germany) and ethanol (anhydrous, >99.5%, Sigma Aldrich, Darmstadt, Germany) for 15 min and dried with argon. Prepared samples were stored in a desiccator until use. Before each test, the samples were cleaned and dried again, similarly.

### 2.3. Morphology and Elemental Composition

The morphology of the produced scaffolds was studied using a FEI Philips XL 40 Environmental Scanning Microscope with an EDS detector, MI, USA. The AZtec software was used to process the results. The average grain size was measured using the line intercept method. Additional elemental analysis was performed using Raman spectroscopy (RamanMicro 200 spectrometer (PerkinElmer, Waltham, MA, USA)). The Raman spectra were registered using a laser with a wavelength of 532 nm, and a maximum power of 20 mW, in the range 50–4400 cm^−1^.

### 2.4. Low-Temperature N_2_ Adsorption Isotherm and Pore Size Distribution

Two iron sponge samples representing different pore sizes and surface areas were analyzed using N_2_ adsorption isotherms at 77 K in the pressure range of 6.58 × 10^−5^ Torr using an Autosorb iQ Station 3 instrument (Quantachrome Instruments, Boynton Beach, Florida, FL, USA). Before adsorption measurements, samples were outgassed for 12 h at 473 K under a high vacuum.

### 2.5. Immersion Enthalpy and Surface Energy Determination

Measurements were carried out using a Tian-Calvet isothermal calorimeter that was constructed in our laboratory [13]. Formamide, deionized water, and n-heptane were used as standard liquids. Each measurement was repeated at least three times. The main assumption of the van Oss–Good–Chaudhury (VGC) model method used in this work is the independence of the interactions of the dispersive and acid–base interactions. Equation (1) describes the enthalpy of immersion for the surface energy components of both the solid surface and the wetting liquid.

(1) $$-h_{i}=-H_{l}+2\left[\sqrt{H_{S}^{LW}\cdot H_{l}^{LW}}+\sqrt{H_{S}^{+}H_{l}^{-}}+\sqrt{H_{S}^{-}H_{l}^{+}}\right]$$

In Equation (1), $h_{i}$ represents the enthalpy change upon immersion (mJ/m^2^); H is the surface enthalpy (mJ/m^2^); s, l are the subscripts, respectively of solid surface and wetting liquid; ^+^, ^−^ subscripts are acidic and basic components of surface energy; and superscript LW is related to the Lifshitz–Van der Waals interactions.

In addition, the second component of the sum in (1) represents the work associated with a particular liquid adhesion, thus one can write the following:

(2) $$-h_{i}=-H_{l}+W_{adh}$$

The energy components of the different probe liquids used in this study were from [14,15].

### 2.6. Corrosion In Vitro 

#### 2.6.1. Static Immersion Test

A static immersion test was performed in Hank’s solution; all samples had a cylinder shape with a 1.5 cm length and 1 cm diameter; each sample was put in 10 mL of solution at 37 °C. The samples were taken out of the soaking after 7, 14, 28, 35, and 56 days, respectively. They were then gently rinsed with distilled water, promptly dried to prevent oxidation, and weighed.

#### 2.6.2. Electrochemical Degradation

Electrochemical degradation was studied via potentiodynamic polarization (PDP) using a potentiostat (BioLogic SP-200). The study was carried out in a standard three-electrode system consisting of a working electrode, a counter-electrode (platinum wire), and a reference electrode (silver/silver chloride electrode). The test was carried out in PBS solution at a constant temperature of 37 ± 1 °C. Before the measurement, the sample was immersed for 120 min in order to measure its open circuit potential (OCP).

The PDP test’s scan potential ranged from −250 mV to +250 mV, relative to the stabilized OCP; measurements were conducted at a 0.167 (mv/s) scan rate.

### 2.7. Cytotoxicity Assessment of the Foams In Vitro

The potential cytotoxicity of the tested scaffolds was assessed using extract testing according to the ISO 10993-5 [16] and ISO 10993-12 [17] norms.

#### 2.7.1. Cell Culture

The in vitro study was conducted using three cell lines: Human Aortic Smooth Muscle Cells (HASMC), Human Umbilical Vein Endothelial Cells (HUVEC) and murine fibroblast cell line L929. HASMC and HUVEC cells, as well as components of their culture media, were obtained from Thermo Fisher Scientific (Waltham, MA, USA), whereas L929 fibroblasts were purchased from American Type Culture Collection (Manassas, VA, USA). HASMC cells were cultivated in Medium 231 supplemented with Smooth Muscle Growth Supplement (SMGS), streptomycin (100 μg/mL) and penicillin (100 IU/mL). HUVEC cells were cultured in Medium 200 containing Low Serum Growth Supplement (LSGS) and antibiotics in the culture flasks pre-coated with Attachment Factor Protein (Thermo Fisher Scientific), which is recommended for use in the HUVEC cell culture since it promotes their attachment and growth. Attachment Factor Protein was also used for the coating of 96-well plates during cytotoxicity assays before cell seeding. L929 fibroblasts were cultured in Roswell Park Memorial Institute (RPMI) 1640 Medium containing 10% fetal bovine serum (FBS) and antibiotics (all reagents from Sigma-Aldrich). All cell lines were cultured at 37 °C in an atmosphere of 5% CO_2_. 

#### 2.7.2. Preparation of Materials and Extracts

The scaffold mats were cut onto 25 mm × 25 mm square specimens and were sterilized using two steps: dipping in 70% ethanol for 20 min and UV light irradiation for 30 min on each side of the samples. After sterilization, the specimens were rinsed with phosphate-buffered saline (pH 7.4; PBS) to remove the residual ethanol. The sterilized specimens were placed into a 6-well plate. The ratio of the area of specimens to the extraction vehicle was 3 cm^2^/mL. For extraction, the samples were placed at 37 °C and 5% CO_2_ for 72 h in the complete culture media appropriate for the respective cell line. The culture media without samples were maintained in the same conditions and used as a non-toxic control. After incubation, the supernatants were centrifuged (2000× *g* force (Relative Centrifugal Force—RCF) for 15 min), followed by sterilization using a 0.22 μm filter. The extraction media were stored at 4 °C no longer than 48 h before use in cell stimulation.

#### 2.7.3. Cell Treatment

To assess the potential cytotoxicity of the tested scaffolds, HASMC, HUVEC and L929 cells were seeded into a 96-well plate at a density of 3 × 10^3^, 5 × 10^3^ and 5 × 10^3^ cells/well, respectively, and pre-incubated for 24 h. Then, the extracts were diluted in an appropriate culture medium using four dilution factors: 1:10, 1:6, 1:3 and 1 (100% of extraction medium), according to the recommendation [18]. The cells were stimulated with the extracts for 24, 48 and 72 h. The control cells were cultured in the corresponding dilution of culture control media, which were prepared similarly to the extracts.

#### 2.7.4. Cell Viability Evaluated Using MTT Assay

The cell viability was assessed using the 3-(4,5-dimethylthiazol-2-yl)-2,5-diphenyltetrazolium bromide (MTT; Sigma Aldrich) assay. This method allows us to determine cell viability based on the reduction of MTT by mitochondrial dehydrogenase to formazan, an enzyme that is active in viable cells. After cell stimulation in a 96-well plate, the cells were rinsed with PBS and 100 μL/well of MTT solution at a final concentration of 0.5 mg/mL in an appropriate culture medium without phenol red was added to each well. The cells were incubated at 37 °C in an incubator for 3 h. Subsequently, the formazan products were dissolved in 100% of dimethylsulfoxide (100 μL/well) by horizontally mixing for 10 min using a microplate shaker. The absorbance values were measured at 570 nm (with a reference wavelength of 630 nm) using a Synergy HT Multi-Mode Microplate Reader (BioTek Instruments, Winooski, VT, USA). The results were presented as a percentage of control cells cultivated in the corresponding dilution of culture control (served as 100%). Blank wells contained extracts or culture control media in a respective dilution without cells. The absorbance values measured in the wells with cells were subtracted from the values estimated for the corresponding blank wells.

#### 2.7.5. Cytotoxicity Assay

The potential cytotoxicity of the specimens was assessed using a CytoTox96^®^ Non-Radioactive Cytotoxicity Assay (Promega Corporation, Madison, WI, USA), which allows the lactate dehydrogenase (LDH) released into the cell culture supernatant during cell lysis to be measured. The positive control for these experiments was the cells stimulated with 0.8% Triton X-100 solution for 45 min at 37 °C. The cells incubated in culture media alone were used as the negative control. After treatment, the cells were rinsed with PBS followed by the LDH assay, which was performed according to the manufacturer’s instructions. The amount of red formazan, which is proportional to the amount of LDH released from dead cells, was measured at 490 nm. The level of LDH released from the cells treated with the extracts, as well as the cells cultured in control media, was presented as a percentage of the cells stimulated with Triton X-100 solution (served as 100%). All calculations were performed after respective blank absorbance subtraction.

#### 2.7.6. Statistical Analysis

The GraphPad Prism 7.0 software (GraphPad Software Inc., La Jolla, CA, USA) was used for statistical analyses. The results were presented as mean ± standard error (SEM) and were analyzed using a one-way analysis of variance (ANOVA) with the post hoc Tukey test. The level of significance was set at *p* < 0.05.

## 3. Results

### 3.1. Morphology and Elemental Composition

**Figure 2:**
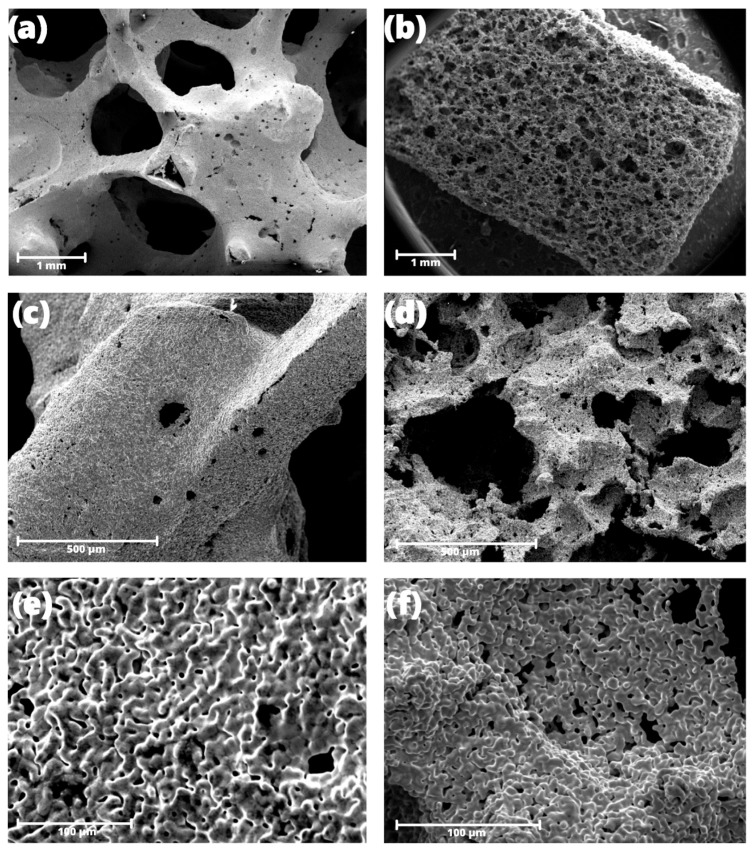
Comparison of the micro-structure of both iron scaffolds. (**a**–**c**) Images show sample Fe01 with increasing magnification. (**d**–**f**) Images showing sample Fe02 with increasing magnification.

The morphology and architecture of the systems are presented using SEM images in Figure 2. The Fe01 sample has bigger pores, with an average size of 0.8–1.6 mm. The average pore size of the Fe02 sample is 0.3–0.16 mm. In the case of the Fe01 sample, smaller pores with a size of 50–60 μm can be seen at higher magnification.

**Figure 3:**
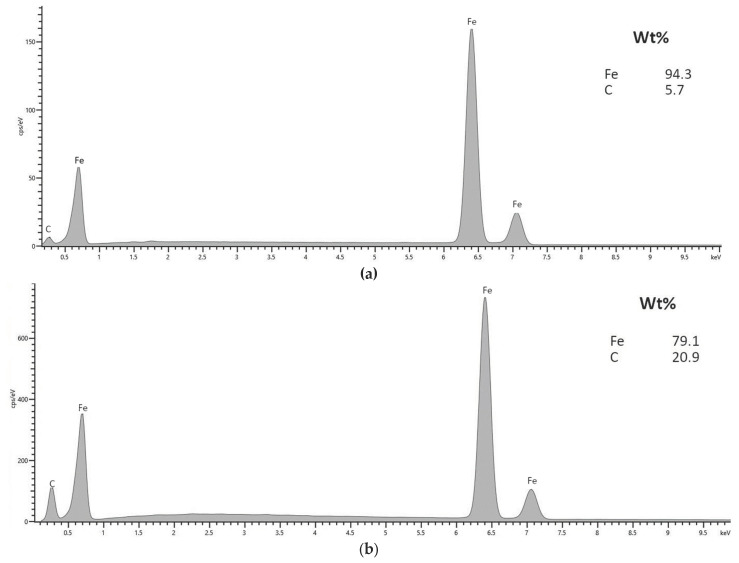
Energy disperses spectroscopy (EDS) spectrum of synthesized material for (**a**) Fe01 and (**b**) Fe02.

The synthesis process requires a high-temperature burn of the polymer template before metal sintering. This is why it is important to ensure that no polymer contamination remains in the resulting material. To test this, we performed the EDS analysis to check both materials’ compositions. The results are in Figure 3.

**Figure 4:**
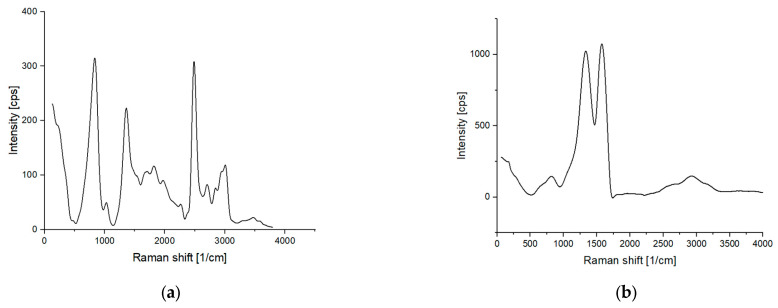
Comparison of Raman spectra taken 24 h after the synthesis process for Fe01 (**a**) and Fe02 (**b**).

Raman spectroscopy was used to determine whether there were any post-process organic residues on the material. The results are shown in Figure 4.

### 3.2. Low-Temperature N_2_ Adsorption Isotherm and Pore Size Distribution

**Figure 5:**
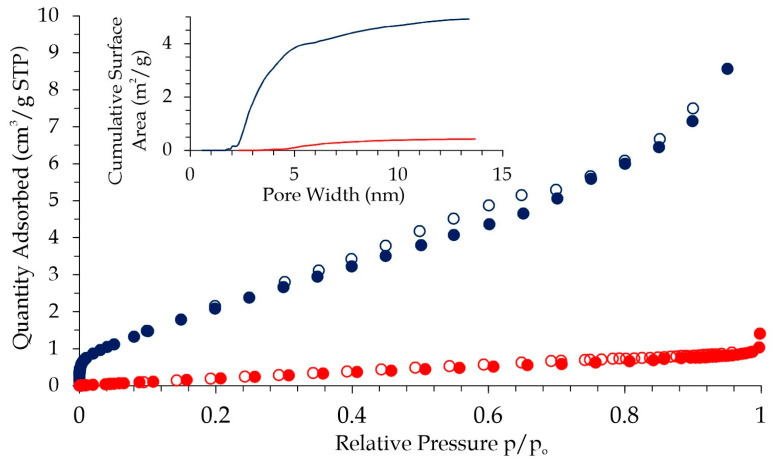
N_2_ adsorption-desorption isotherm for Fe01 (red) and Fe02 (blue color) samples.

The low-temperature N_2_ adsorption isotherm was performed for both analyzed samples, and the results are in Figure 5. These isotherms are in the second type characteristic for macro-porous adsorbents, differing drastically in their surface area and pore volume [19]. 

Interestingly, a mono-layer formation, i.e., a “knee” in the low-pressure range, was observed only for Fe02 material. This is the consequence of mesopores in the range of 2–4 nm, which can develop a surface area of up to 4.4 m^2^/g for a Fe02 sample.

A further increase in the N_2_ pressure caused the amount of adsorbed N_2_ to increase exponentially. The overall adsorption mechanism of these materials is based on the fact that once the small droplet of adsorbate nucleates, further adsorption occurs more easily due to adsorbate–adsorbate interactions being higher than adsorbate–adsorbent interactions.

### 3.3. Immersion Enthalpy and Surface Energy Determination

Because the measured surface area of the Fe01 sample was below 0.5 m^2^/g, the surface energy determination was performed only for the Fe02. In the case of Fe01, the results were infinitesimally small.

The characterization of the surface energy components using the van Oss–Good–Chaudhury (VGC) approximation is shown in Table 1. The used model easily separates the Lifshitz–Van der Waals component of the surface energy, *H_S_^LW^*, from polar terms, i.e., the acidic, *H_S_^+^*, and the basic, *H_S_^−^*, components, allowing the calculation of the total surface energy, *H_S_^T^*. In addition, the separation of the acidic and basic components of the surface free energy due to the ionic nature of bonds present on the surface provides information about the interactions of adsorbents with molecules on the surface. The enthalpies of immersion in different probe liquids, namely water, *n*-hexane, and formamide (*h_i_*), were measured.

The results collected revile the acidic character of the Fe-based material. The calculated *H_S_^+^* is 27 times larger than *H_S_^−^*. Moreover, the electrostatic interactions dominate here over the non-polar ones. A high water work of adhesion, i.e., 2.12 J/m^2^, as well as a high total surface enthalpy of 3.45 J/m^2^, were determined.

### 3.4. Corrosion In Vitro

**Figure 6:**
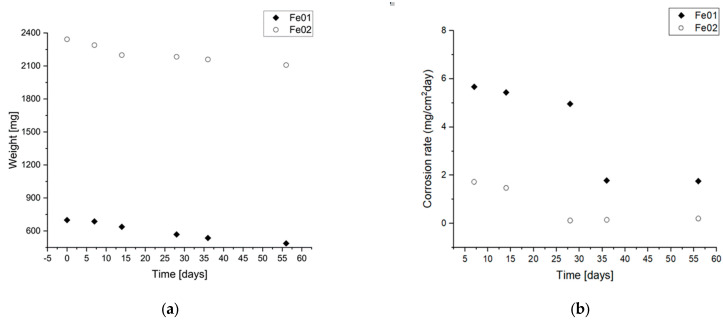
(**a**) Graph showing the loss of weight over time during immersion in a pseudo-physiological fluid at 37 °C, (**b**) Graph showing corrosion rate calculated form Equation (3).

Figure 6 presents the weight loss of the samples within 2 months, which can be named as corrosion speed [20,21]. Corrosion rates were calculated from the mass loss measurements (Figure 6a) using the following formula:

(3) CR=M/At

where M is the mass loss [mg], A is the exposed surface area [cm^2^], and *t* is the exposure time [days].

The exposed surface was 0.5 m^2^/g for Fe01 and 4.4 m^2^/g for Fe02 from the low-temperature N_2_ adsorption described in 3.2. In the first month, the weight loss was 18.7% for Fe01 and 6.8% for Fe02. For Fe01, it amounted to 30.3% weight loss within two months, while for Fe02 it was 9.98%.

### 3.5. Electrochemical Degradation

**Figure 7:**
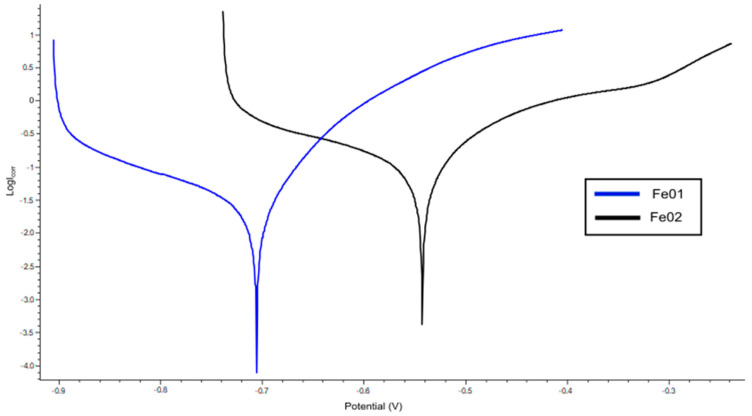
Polarization curves of obtained samples, where (a-blue) Fe01; (b-black) Fe02.

The electrochemical test was carried out in accordance with the procedure described in Section 2.6.2. The obtained potentiodynamic curves are presented in the graph in Figure 7. Based on the tests carried out, the data for both samples included in Table 2 were established.

### 3.6. Tested Scaffolds Induced Cytotoxic Effect on the Cells

**Figure 8:**
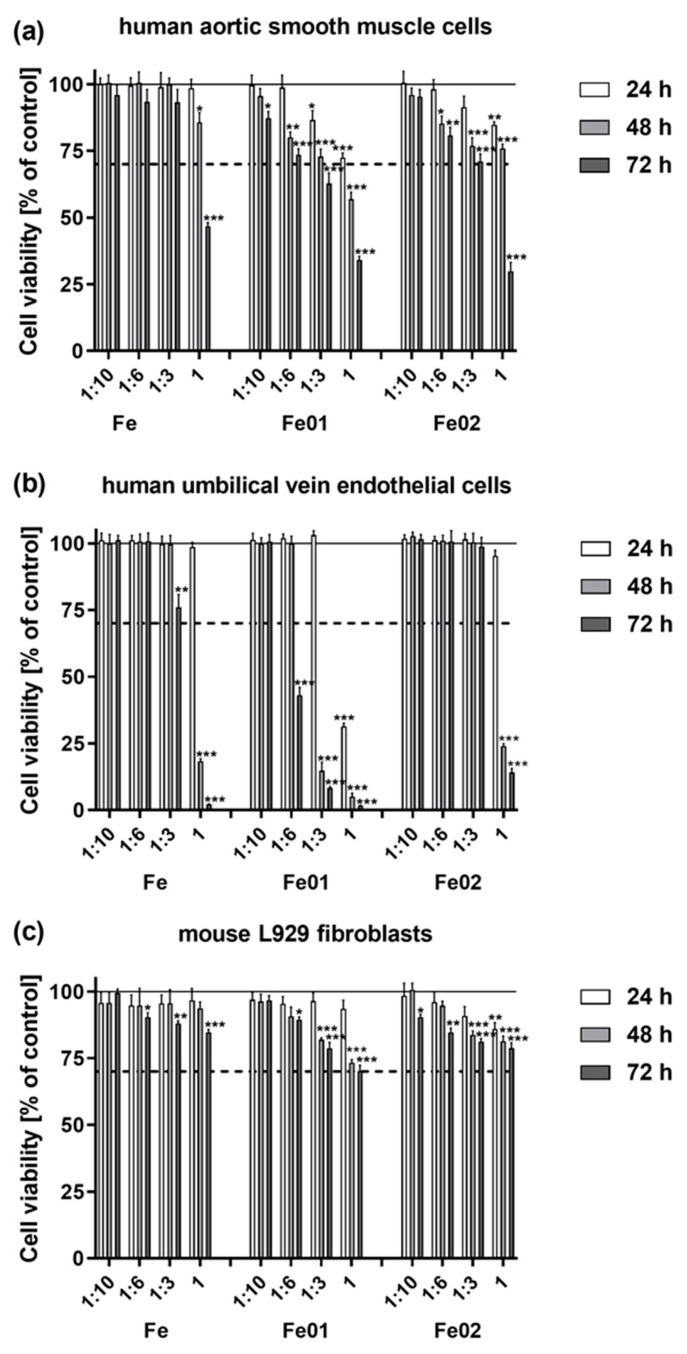
The viability of human aortic smooth muscle cells (**a**), human umbilical vein endothelial cells (**b**) and murine fibroblast cell line L929 (**c**) stimulated with the extracts derived from the tested scaffolds. Cells were incubated for 24, 48 and 72 h with the extracts diluted in an appropriate culture medium using four dilution factors: 1:10, 1:6, 1:3 and 1. Cell viability was presented as a percentage ± S.E.M. of the control cells. Asterisks show statistical differences between the control cells (served as 100%; solid line) and the cells treated with the extracts (*** *p* < 0.001; ** *p* < 0.01; * *p* < 0.05). The dashed line presents the potential cytotoxicity when the cell viability decreases below 70% according to ISO norms.

The cytotoxic effect of the tested scaffolds was evaluated using three cell lines: human aortic smooth muscle cells (HASMC), human umbilical vein endothelial cells (HUVEC) and murine L929 fibroblasts, and two assays that measured cell viability (MTT assay) and the level of cell death (LDH assay) were used. As seen in Figure 8, the extracts derived from the Fe01 scaffolds, as well as the Fe02 specimens, significantly reduced the viability of all the tested cell lines in a time-dependent and dilution-dependent manner. The least cytotoxic effect was observed for L929 fibroblasts (Figure 8c) when the cell viability was always above 70%, including the treatment of cells with undiluted extracts for 72 h (70.1 ± 2.3% for Fe01 specimens and 78.8 ± 2.0% for Fe02 samples, respectively). These results indicated that the tested scaffolds cannot be considered as cytotoxic materials for L929 cells, according to the ISO standards. In the case of HASMC cell treatment, their survival rate was reduced below 70% when the cells were incubated with the undiluted extract derived from Fe01 scaffolds for 48 and 72 h (56.9 ± 2.6% and 34.1 ± 1.4%, respectively), and after stimulation with this extract diluted at 1:3 for 72 h (62.8 ± 3.9%). A similar cytotoxic effect was observed for Fe02 specimens only upon the stimulation of HASMC cells with undiluted extract for 72 h (29.9 ± 3.3%) (Figure 8a). HUVEC cells were found to be the most sensitive to the stimulation with the extract derived from Fe01 samples since their viability was significantly decreased below 70% already after treatment with the extract diluted at 1:6 for 72 h (43.1 ± 2.9%), as well as upon incubation with the undiluted extract for 24 h (31.4 ± 1.2%). In response to the challenge with the extract derived from Fe02 scaffolds, the cytotoxic effect on HUVEC cells was observed for the undiluted extract after 48 and 72 h of incubation time (24.0 ± 0.9% and 14.1 ± 1.5%, respectively) (Figure 8b). In contrast, the incubation of cells with the undiluted extracts derived from Fe samples decreased the cell survival below 70% in the case of HASMC cells treated for 72 h (46.8 ± 1.4%), and HUVEC cells incubated for either 48 or 72 h (18.3 ± 1.0% and 2.2 ± 0.1%, respectively).

**Figure 9:**
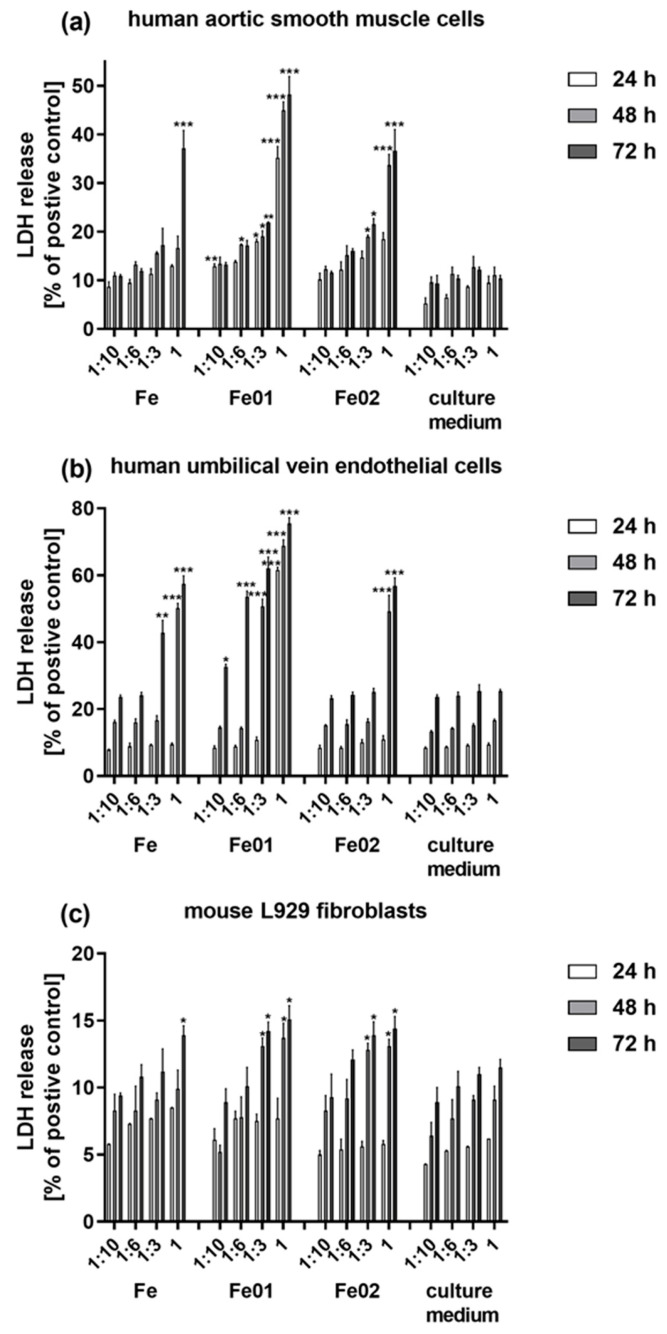
Lactate dehydrogenase (LDH) release from human aortic smooth muscle cells (**a**), human umbilical vein endothelial cells (**b**) and murine fibroblast cell line L929 (**c**) stimulated with the extracts derived from the tested scaffolds. Cells were incubated for 24, 48 and 72 h with the extracts diluted in an appropriate culture medium using four dilution factors: 1:10, 1:6, 1:3 and 1. The results are expressed as the percentage ± S.E.M. of the cells stimulated with 0.8% Triton X-100 solution (served as 100%). Asterisks show statistically significant differences between the cells cultured in control media (culture medium) and the cells incubated with the corresponding diluted extracts (*** *p* < 0.001; ** *p* < 0.01; * *p* < 0.05).

The LDH release assay supported the results of the MTT test, indicating that a time-dependent and dilution-dependent increase in LDH leakage was observed for all cell lines stimulated with the extracts derived from the scaffolds. The time-dependent effect was also observed for the HUVEC and L929 cells cultivated in the culture control media, however, the measured levels of LDH were significantly lower in comparison with the cells treated with the extracts (Figure 8). Compared with the positive control, which determines the 100% cytotoxic effect on cells, the highest level of cytotoxicity was observed for HUVEC cells treated for 72 h with the undiluted extract derived from Fe01 scaffolds (75.5 ± 1.7%), Fe samples (57.4 ± 2.4%) and Fe02 specimens (56.8 ± 2.4%). Moreover, as seen in Figure 9b, the extracts derived from Fe01 scaffolds induced also a significantly high level of LDH release upon HUVEC cell stimulation with the extract diluted at either 1:6 or 1:3. The cytotoxicity measured for HUVEC cells cultured in the extracts from Fe samples was similar to that observed upon treatment with the Fe02 specimens-derived extract. Among all the tested cells lines, the weakest cytotoxic effect was observed against L929 fibroblasts, when the level of LDH release did not exceed 15.1 ± 1.0% (value measured for the cells incubated with the undiluted extract from Fe01 samples for 72 h; *p* < 0.05) (Figure 9c). In the case of HASMC cell treatment, the highest cytotoxicity was noticed for the cells cultured in the undiluted extracts derived from Fe01 specimens for 48 and 72 h (45.0 ± 1.7% and 48.2 ± 3.7%, respectively) (Figure 9a).

## 4. Discussion

By comparing the SEM images in Figure 2, the fundamental differences between Fe01 and Fe02 can be observed. Fe01 has large, open pores all over the surface, forming the so-called cell windows [12,22], i.e., interconnected, intersected, and creating a network of open space structures in the material. The sizes of these windows are in the range of 0.8–1.6 mm, which fully corresponds to the pore size of the polyurethane template. Interestingly, the images clearly show the second type of pores (Figure 2b), much smaller ones randomly distributed over the entire surface of the sample. Their size is in the range of 150–25 μm, so they are approximately 10 times smaller than the cell windows size. This type of pore does not appear on the template used for the synthesis. Most likely, they arise when, during the evaporation of the PVA solution, iron particles combine into larger agglomerations, leaving free space in some places. During sintering, this aggregation is fixed, and micro-pores remain in the finished sample. The mere presence of micro-pores does not have to have a negative effect on the material as they increase the contact surface with the physiological fluid and can act as local centers, where degradation will begin. To check this theory, it is necessary to carry out an immersion test in a pseudo-physiological fluid and take SEM pictures at a specific time interval. This way, we could determine where and when the corrosion process begins.

The cracks in the strut appearing in the structure are problematic for the architecture set by the template in Figure 2a. The strut thickness is approximately 50–250 μm, while the crack depth is up to 2–20 μm, which can significantly weaken the entire structure and decrease its mechanical properties.

The pores of the Fe02 sample (Figure 2d) are much smaller (50–60 μm) and do not form such an extensive network of windows in the material as in the Fe01 sample, although these are also open pores. Due to the tiny “main” pores, it is difficult to determine whether there are secondary micro-pores as in Fe01. Still, it can be seen that the architecture of the Fe02 material is much more uniform, and there are no significant cracks or voids. 

The melamine sponge used as a template for the preparation of Fe02 is much more absorbent, which means that when preparing the material, it is easier to supply a large amount of PVA solution with iron inside. For this reason, the internal structure of Fe02 is perhaps more homogeneous and reproducible. However, this can have positive connotations because, thanks to the dense network of beams, the mechanical properties of this material should last longer, and the likelihood of larger pieces of material detaching is much lower. We will explore this in future research.

Considering the application, the first material (Fe01) may be more applicable in bone implant technology, where a porosity of the order of 250–300 μm is necessary for the growth of tissue [23]. The smallest porosity enabling vascularization of scaffolds and bone ingrowth is 120 μm, which means that Fe02 would probably not be used in the regeneration of bone defects [24]; perhaps it would have more significant potential in the use of cardiovascular implants, where porous iron also applies [25]. 

By comparing the EDS analyses for both samples (Figure 3) it can be seen that there is a strong iron signal and a smaller signal from carbon. The material based on melamine sponge (Fe02) has a higher carbon percentage (20.9%) than the Fe01 sample (5.7%). These are remnants of the template that were not removed during the process. Carbon, as a fully biocompatible material, should not affect the cytotoxicity of the material, but it may have some effect on its corrosion. Whatis more, adding carbon should improve the material’s mechanical strength, which is a well-known relationship used, among others, in steel production. However, the presence of carbon at the level of 1/5 of the total material in Fe02 can be a problem due to the difficulty of controlling and predicting the final composition of the material. In subsequent studies, it is necessary to examine whether the distribution of carbon in the material is regular and whether it form any clusters or phases or not.

Theoretically, at 1200 °C, all organic residues should be reduced to carbon or removed in gaseous form. The Raman spectrum (Figure 4a) confirms this theory in the case of the Fe02 material, where the main oscillating bands are characteristics for carbon: 1379 cm^−1^ comes from D band and 1616 cm^−1^ comes from G band [26]. The fact that the intensity of the D and G bands is very similar confirms that we are dealing with disordered carbon from post-reaction residues [27]. This is consistent with the results from the EDS.

The analysis of the Raman spectra in Figure 4b does not confirm this in the case of the Fe01 material, where a mixture of organic compounds was found in the sample. While producing samples on a polyurethane template, process residues form an oily, yellow liquid that is deposited on the cool parts of the reactor. Some of these residues are most likely absorbed by the sample during the cooling phase, and the material is contaminated.

These impurities can primarily affect the biological properties of the material. Removing this problem during the sintering process is a big challenge for future investigations. Therefore, a melamine sponge that leaves mainly carbon behind is a much better choice in this comparison. The analysis of the literature reports showed a lack of information regarding the analysis of the Raman spectra of materials produced by the method we proposed. Thus, it is difficult to compare the results obtained with other studies and to conclude whether this is a problem with our reactor or whether it is due to other factors affecting the contamination of the manufactured template. This result makes it worthwhile to specify in advance and determine the mechanism of its degradation and identify its decomposition products when selecting a polymer matrix.

To determine the surface area of pores, adsorption measurements were carried out. These investigations revealed that the sample Fe02 had a higher surface area relative to sample Fe01, which was caused using different starting materials. Sample Fe01 was produced from a polyurethane template, and sample Fe02 was from a melamine template. This shows that the type of templating used influences the specific surface area value. Measuring the wetting angle for samples with such large pores is not feasible; thus, determining the surface free energy is impossible using the Owens–Wendt method. Therefore, as in our earlier work, calorimetric measurements were performed. The enthalpies of immersion in different probe liquids were measured to characterize the surface energy components using the van Oss–Good–Chaudhury (VGC) approximation. Compared to our previous results, lowering the pore size causes an increase in SA and an additional capillary effect, causing a rise in the work of water adhesion. The high value of the parameter results in the good wettability of the material in a natural tissue environment.

To determine the degradation rate of the material and thus its suitability for biomedical purposes as biodegradable, we carried out corrosion tests. Mass loss during corrosion under static conditions is shown in Figure 6. Already in the first week of immersion in Hank’s solution, both samples degraded. The beginning of degradation was visible, as they began to turn reddish-brown locally and, over time, completely changed color, dyeing also the solution.

Comparing the corrosion rate from month to month, we can conclude that corrosion is initially the fastest and slows down over time; in the case of sample Fe01, in the first month, it degraded at 18.7%, which is 61.72% of all degradation, and in the case of Fe02, in the first month, it degraded 6.90% which amounted to 69.14% of all degradation. The difference is slight, but undeniably, the material degraded faster in the first month than in the second. The change in this rate may be due to the accumulation of degradation products that have settled over the entire surface of the sample, thereby limiting the material’s contact with the pseudo-physiological fluid and making corrosion difficult. It should be noted that during the entire test, the corrosion rate of Fe01 was approximately three times greater than Fe02. This happens due to the greater porosity of Fe01, where the cell windows network means that almost the entire material is in constant contact with Hank’s solution from the beginning. In the case of Fe02, the surface area is smaller, which slows down degradation.

Analyzing the results of electrochemical tests (Figure 7), we conclude that the Fe01 material corrodes faster because the polarization curve shifts into more active potential values. This confirms the literature reports, which show that increasing the contact area increases the number of Fe ions involved in the electrochemical reaction, which results in an accelerated corrosion process [28]. The polarization curves have a shape typical for metal corrosion with active dissolution. Comparing this result with the iron-based porous material obtained by other teams [11,29], we see that both of our materials degrade slightly faster, which is desirable. Further modifications of the corrosion rate are possible through additives or coatings [30,31,32]. 

To fully assess the material’s potential for application as an implant, it was necessary to perform cytotoxicity studies. The biocompatibility of the tested iron scaffolds was estimated, according to the ISO 10993-5 and ISO 10993-12 standards, using an extract method to evaluate the potential cytotoxicity of any leachable products from the materials. It is well established that the toxicity of metallic materials, including iron, is governed mainly by the toxicity of the released metallic ions and material degradation products [33]. Although the ISO 10993-12 standard allows for the extraction of materials only for 24 h, we have decided to prolong the extraction time up to 72 h, since the tested scaffolds may have long-term contact with the biological microenvironment after implantation.

In the present study, the potential cytotoxicity of the scaffolds was evaluated using three cell lines: human aortic smooth muscle cells (HASMC), human umbilical vein endothelial cells (HUVEC) and murine fibroblast cell line L929, which represent cells essential for the evaluation of the biocompatibility of material, which can be used in cardiovascular implants. Firstly, the stent is mainly in contact with endothelial and smooth muscle cells during its lifetime, simultaneously during the initial wound-healing phase, or exclusively with smooth muscle cells after neointima formation [34]. HASMC cells are one of the vascular smooth muscle cell types that is the fundamental cellular component and primarily govern structural integrity and regulate the vascular tone of the blood vessel wall [35]. HUVEC cells are very often used in research related to the function of blood vessel endothelium. Moreover, endothelial cells are responsible for the proper functioning of the coronary arteries, tightly controlling the migration and proliferation of smooth muscle cells and inhibiting platelet activation inside the blood. Rapid recovery of endothelium at the coronary intervention site is, therefore, considered to be a critical factor in the healing process of the arterial walls [36,37]. Finally, L929 fibroblasts are widely used in the assessment of the potential cytotoxicity of biomaterials in vitro [38] and the integrity of the peri-implant soft-tissue seal is crucial for maintaining peri-implant tissue health [39].

Generally, in the present study, the results from the MTT and LDH assays showed that the extracts derived from Fe01 and Fe02 scaffolds had a more toxic effect on HASMS and HUVEC cells than the extracts from the Fe references specimens, which occurred in a time-dependent and dilution-dependent manner. However, these iron reference samples also induced some level of cytotoxicity against these two cell lines. In contrast, the viability level of L929 fibroblasts stimulated with all tested extracts was always above 70%, which indicates that the tested scaffolds cannot be considered as cytotoxic materials for L929 cells, according to the ISO standards. In the case of the treatment of HASMC cells, a significantly reduced cell viability below 70% was observed only during incubation with the undiluted extracts. It should be emphasized that in a clinical condition, the potential cytotoxicity of the tested material may be even lower since the extracts usually are diluted by the surrounding tissue fluid [40,41]. Among the tested cell lines, HUVEC cells were found to be the most sensitive to the stimulation with the extract derived from Fe01 scaffolds, when the cytotoxic effect was observed not only for undiluted extracts but also the extracts diluted even 1:6 after 72 h of incubation. In contrast, in response to the challenge with the extract derived from Fe02 specimens, the cytotoxic effect on HUVEC cells was observed for the undiluted extract only. Several previous studies have reported the in vitro cytotoxicity of different iron-based materials for endothelial cells [42,43], smooth muscle cells [44], and L929 fibroblasts [30]. These results have shown that the cytotoxicity level mainly depends on ferrous ion concentrations released from the materials. Moreover, since it is well established that reactive oxygen species (ROS) are generated via the catalytic decomposition of hydrogen peroxide by ferrous ions leading to the Fenton reaction-mediated cell cytotoxicity [45], we presume that this phenomenon can be responsible for the cytotoxic effect induced by the tested materials. However, additional studies are required to confirm this assumption. Taking into consideration the results from our cytotoxicity studies performed for all tested cell lines, it seems to be clear that the Fe02 scaffold is a better candidate for the production of cardiovascular scaffolds than the Fe01 specimen, despite the fact that both types of scaffolds show some cytotoxic properties. 

## 5. Conclusions

Summing up, the presented results lead to the following conclusions: 

Firstly, the template morphology strongly influences the corrosion path of the obtained material. With the porosity increasing and thus the surface area, the corrosion rate increases significantly. However, it is important to note that while a substantial acceleration of iron template corrosion is desired, this effect is associated with the cytotoxicity of the material. As the secretion of corrosion products increases, the viability of cell lines decreases significantly, which is an unfavorable effect. Therefore, there is a need to balance the corrosion rate and biocompatibility.

Raman spectroscopy enabled the detection of organic species in the Fe01 sample, which are the thermal decomposition products of the polyurethane template. In the next stage of our research, we will develop a method for removing the decomposition products of the polyurethane template.

Next, the material undergoes testing for mechanical properties. In the future, we plan to modify the obtained materials by doping iron with other metals to improve the biocompatibility of the systems.

## Figures and Tables

**Table 1:** The surface energy components, and the work of adhesion, *W_adh_*, of Fe foam to water.

h_water_ [J/m^2^]	h_n-heptane_ [J/m^2^]	h_formamide_ [J/m^2^]	$H_{S}^{LW}$ [J/m^2^]	$H_{S}^{+}$ [J/m^2^]	$H_{S}^{-}$ [J/m^2^]	$H_{S}^{T}$ [J/m^2^]	$W_{adh}$ [J/m^2^]
−1.763 (0.21)	−0.352 (0.0201)	−1.951 (0.141)	0.760 (0.263)	13.669 (0.382)	0.530 (0.089)	3.453 (0.029)	2.123 (0.042)

**Table 2:** Corrosion parameters for both samples.

	E corr [mV]	I corr [μA]
Fe01	705,739	16,951
Fe02	542,397	80,490

## References

[1] Mani G., Feldman M.D., Patel D., Agrawal C.M. (2007). Coronary Stents: A Materials Perspective. Biomaterials.

[2] Chesta F., Rizvi Z.H., Oberoi M., Buttar N. (2020). The Role of Stenting in Patients with Variceal Bleeding. Tech. Innov. Gastrointest. Endosc..

[3] Law M.A., Shamszad P., Nugent A.W., Justino H., Breinholt J.P., Mullins C.E., Ing F.F. (2010). Pulmonary Artery Stents: Long-Term Follow-Up. Catheter. Cardiovasc. Interv..

[4] Zheng Y., Yang H. (2020). Manufacturing of Cardiovascular Stents. Metallic Biomaterials Processing and Medical Device Manufacturing.

[5] Bowen P.K., Shearier E.R., Zhao S., Guillory R.J., Zhao F., Goldman J., Drelich J.W. (2016). Biodegradable Metals for Cardiovascular Stents: From Clinical Concerns to Recent Zn-Alloys. Adv. Healthc. Mater..

[6] Tong X., Wang H., Zhu L., Han Y., Wang K., Li Y., Ma J., Lin J., Wen C., Huang S. (2022). A Biodegradable in Situ Zn–Mg2Ge Composite for Bone-Implant Applications. Acta Biomater..

[7] Zartner P., Cesnjevar R., Singer H., Weyand M. (2005). First Successful Implantation of a Biodegradable Metal Stent into the Left Pulmonary Artery of a Preterm Baby. Catheter. Cardiovasc. Interv..

[8] Kaushik V., Nithish Kumar B., Sakthi Kumar S., Vignesh M. (2022). Magnesium Role in Additive Manufacturing of Biomedical Implants—Challenges and Opportunities. Addit. Manuf..

[9] Gąsior G., Szczepański J., Radtke A. (2021). Biodegradable Iron-Based Materials—What Was Done and What More Can Be Done?. Materials.

[10] Salama M., Vaz M.F., Colaço R., Santos C., Carmezim M. (2022). Biodegradable Iron and Porous Iron: Mechanical Properties, Degradation Behaviour, Manufacturing Routes and Biomedical Applications. J. Funct. Biomater..

[11] Hrubovčáková M., Kupková M., Džupon M. (2016). Fe and Fe-P Foam for Biodegradable Bone Replacement Material: Morphology, Corrosion Behaviour, and Mechanical Properties. Adv. Mater. Sci. Eng..

[12] Oriňaková R., Gorejová R., Králová Z.O., Petráková M., Oriňak A. (2021). Novel Trends and Recent Progress on Preparation Methods of Biodegradable Metallic Foams for Biomedicine: A Review. J. Mater. Sci..

[13] Wiśniewski M., Rychlicki G., Arcimowicz A. (2010). Experimental and Theoretical Estimations of the Polar Force Contributions to the Heat of Immersion of Carbon Nanotubes. Chem. Phys. Lett..

[14] Grodzicka M., Gąsior G., Wiśniewski M., Bartmański M., Radtke A. (2022). A Simple Replica Method as the Way to Obtain a Morphologically and Mechanically Bone-like Iron-Based Biodegradable Material. Materials.

[15] Douillard J.-M., Salles F., Henry M., Malandrini H., Clauss F. (2007). Surface Energy of Talc and Chlorite: Comparison between Electronegativity Calculation and Immersion Results. J. Colloid Interface Sci..

[16] (2009). Biological Evaluation of Medical Devices—Part 5: Tests for In Vitro Cytotoxicity.

[17] (2021). Biological Evaluation of Medical Devices—Part 12: Sample Preparation and Reference Materials.

[18] Kubásek J., Vojtěch D., Jablonská E., Pospíšilová I., Lipov J., Ruml T. (2016). Structure, Mechanical Characteristics and in Vitro Degradation, Cytotoxicity, Genotoxicity and Mutagenicity of Novel Biodegradable Zn–Mg Alloys. Mater. Sci. Eng. C.

[19] Lowell S., Shields J.E. (1991). Adsorption Isotherms. Powder Surface Area and Porosity.

[20] Birbilis N., Holloway L.J. (2007). Use of the Time Constant to Detect Corrosion Speed in Reinforced Concrete Structures. Cem. Concr. Compos..

[21] Sriramadasu R.C., Lu Y., Banerjee S., Sriramula S. (2022). Advances in Corrosion Monitoring of Reinforced Concrete Using Active and Passive Sensing Approaches. The Rise of Smart Cities.

[22] Han Y., Choi J., Kim H.-S., Kim H., Park J. (2013). Control of Pore and Window Size of Ceramic Foams with Tri-Modal Pore Structure: Influence of Agar Concentration. Mater. Lett..

[23] Karageorgiou V., Kaplan D. (2005). Porosity of 3D Biomaterial Scaffolds and Osteogenesis. Biomaterials.

[24] Murphy C.M., Haugh M.G., O’Brien F.J. (2010). The Effect of Mean Pore Size on Cell Attachment, Proliferation and Migration in Collagen–Glycosaminoglycan Scaffolds for Bone Tissue Engineering. Biomaterials.

[25] Sharma P., Jain K.G., Pandey P.M., Mohanty S. (2020). In Vitro Degradation Behaviour, Cytocompatibility and Hemocompatibility of Topologically Ordered Porous Iron Scaffold Prepared Using 3D Printing and Pressureless Microwave Sintering. Mater. Sci. Eng. C.

[26] Li Z., Deng L., Kinloch I.A., Young R.J. (2023). Raman Spectroscopy of Carbon Materials and Their Composites: Graphene, Nanotubes and Fibres. Prog. Mater. Sci..

[27] Puech P., Kandara M., Paredes G., Moulin L., Weiss-Hortala E., Kundu A., Ratel-Ramond N., Plewa J.-M., Pellenq R., Monthioux M. (2019). Analyzing the Raman Spectra of Graphenic Carbon Materials from Kerogens to Nanotubes: What Type of Information Can Be Extracted from Defect Bands?. C—J. Carbon Res..

[28] Sharma P., Pandey P.M. (2019). Rapid Manufacturing of Biodegradable Pure Iron Scaffold Using Amalgamation of Three-Dimensional Printing and Pressureless Microwave Sintering. Proc. Inst. Mech. Eng. J. Mech. Eng. Sci..

[29] Haverová L., Oriňaková R., Oriňak A., Gorejová R., Baláž M., Vanýsek P., Kupková M., Hrubovčáková M., Mudroň P., Radoňák J. (2018). An In Vitro Corrosion Study of Open Cell Iron Structures with PEG Coating for Bone Replacement Applications. Metals.

[30] Liu B., Zheng Y.F. (2011). Effects of Alloying Elements (Mn, Co, Al, W, Sn, B, C and S) on Biodegradability and in Vitro Biocompatibility of Pure Iron. Acta Biomater..

[31] Huang T., Zheng Y., Han Y. (2016). Accelerating Degradation Rate of Pure Iron by Zinc Ion Implantation. Regen. Biomater..

[32] Huang S., Ulloa A., Nauman E., Stanciu L. (2020). Collagen Coating Effects on Fe–Mn Bioresorbable Alloys. J. Orthop. Res..

[33] Moravej M., Mantovani D. (2011). Biodegradable Metals for Cardiovascular Stent Application: Interests and New Opportunities. Int. J. Mol. Sci..

[34] Scarcello E., Lison D. (2019). Are Fe-Based Stenting Materials Biocompatible? A Critical Review of In Vitro and In Vivo Studies. J. Funct. Biomater..

[35] Zhao Y., Zang G., Yin T., Ma X., Zhou L., Wu L., Daniel R., Wang Y., Qiu J., Wang G. (2021). A Novel Mechanism of Inhibiting In-Stent Restenosis with Arsenic Trioxide Drug-Eluting Stent: Enhancing Contractile Phenotype of Vascular Smooth Muscle Cells via YAP Pathway. Bioact. Mater..

[36] Ceylan H., Tekinay A.B., Guler M.O. (2011). Selective Adhesion and Growth of Vascular Endothelial Cells on Bioactive Peptide Nanofiber Functionalized Stainless Steel Surface. Biomaterials.

[37] Yau J.W., Teoh H., Verma S. (2015). Endothelial Cell Control of Thrombosis. BMC Cardiovasc. Disord..

[38] Thrivikraman G., Madras G., Basu B. (2014). In Vitro/In Vivo Assessment and Mechanisms of Toxicity of Bioceramic Materials and Its Wear Particulates. RSC Adv..

[39] Furuhashi A., Ayukawa Y., Atsuta I., Okawachi H., Koyano K. (2012). The Difference of Fibroblast Behavior on Titanium Substrata with Different Surface Characteristics. Odontology.

[40] Bae W.-J., Chang S.-W., Lee S.-I., Kum K.-Y., Bae K.-S., Kim E.-C. (2010). Human Periodontal Ligament Cell Response to a Newly Developed Calcium Phosphate–Based Root Canal Sealer. J. Endod..

[41] Gomes M.E., Reis R.L., Cunha A.M., Blitterswijk C.A., de Bruijn J.D. (2001). Cytocompatibility and Response of Osteoblastic-like Cells to Starch-Based Polymers: Effect of Several Additives and Processing Conditions. Biomaterials.

[42] He H., Qiao Y., Zhou Q., Wang Z., Chen X., Liu D., Yin D., He M. (2019). Iron Overload Damages the Endothelial Mitochondria *via* the ROS/ADMA/DDAHII/ENOS/NO Pathway. Oxid. Med. Cell. Longev..

[43] Zhu S., Huang N., Xu L., Zhang Y., Liu H., Sun H., Leng Y. (2009). Biocompatibility of Pure Iron: In Vitro Assessment of Degradation Kinetics and Cytotoxicity on Endothelial Cells. Mater. Sci. Eng. C.

[44] Moravej M., Purnama A., Fiset M., Couet J., Mantovani D. (2010). Electroformed Pure Iron as a New Biomaterial for Degradable Stents: In Vitro Degradation and Preliminary Cell Viability Studies. Acta Biomater..

[45] Igarashi K., Shoji Y., Sekine-Suzuki E., Ueno M., Matsumoto K., Nakanishi I., Fukui K. (2022). Importance of Locations of Iron Ions to Elicit Cytotoxicity Induced by a Fenton-Type Reaction. Cancers.

